# Therapeutic use of ayahuasca: a review of the evidence of its use in approaching depressive disorders

**DOI:** 10.1192/j.eurpsy.2022.1843

**Published:** 2022-09-01

**Authors:** G. Simões, S. Jesus, R. Silva

**Affiliations:** Baixo Vouga Hospital Centre - EPE, Psychiatry And Mental Health Department, Aveiro, Portugal

**Keywords:** Ayahuasca, therapeutical use, depressive disorders, Pharmacology

## Abstract

**Introduction:**

Ayahuasca (AYA) is a psychotropic plant from South America used for religious purposes by indigenous people of the Amazon. Increasing evidence indicates that AYA may have therapeutic potential in the treatment of mental health disorders like depression – a common life-disrupting, highly recurrent disorder – that is among the leading causes of disability worldwide.

**Objectives:**

The aim of this exploratory study is to gather and assess scientific evidence about clinical effects of AYA in the treatment and symptomatological expression of patients with depression.

**Methods:**

A literature research was conducted on PubMed, starting from the MeSH terms: “Banisteriopsis” and “Depression”. Results corresponding to investigations using AYA, and based on an adult population with depressive disorders, were selected for our analysis.

**Results:**

The research provided 8 results, of which 6 met the defined criteria. Different types of studies with variable samples were considered, including retrospective and prospective observational studies, meta-analysis and a narrative review. Overall, evidence about the use of AYA in depressive disorders is associated to reductions in depression scales, to significant antidepressant effects and in mediating improvement of grief symptoms. AYA administration increased introspection and positive mood, self-acceptance, empathy, openness and potentiated improvements in emotional processing. The underlying potential mechanisms, adverse effects and the current limitations related to its study and use are analysed and discussed.

**Conclusions:**

The use of AYA in depression shows promising results that should be further explored in controlled trials with larger sample sizes, in order to better evaluate its clinical effects, safety profile and related short and long-term effects.

**Disclosure:**

No significant relationships.

